# CRISPR-MFH: A Lightweight Hybrid Deep Learning Framework with Multi-Feature Encoding for Improved CRISPR-Cas9 Off-Target Prediction

**DOI:** 10.3390/genes16040387

**Published:** 2025-03-28

**Authors:** Yanyi Zheng, Quan Zou, Jian Li, Yanpeng Yang

**Affiliations:** 1 College of Landscape Architecture, Beijing Forestry University, Beijing 100083, China; zhengyanyi@bjfu.edu.cn; 2 Institute of Fundamental and Frontier Sciences, University of Electronic Science and Technology of China, Chengdu 610054, China; zouquan@nclab.net; 3 Yangtze Delta Region Institute (Quzhou), University of Electronic Science and Technology of China, Quzhou 324000, China; 4 School of Mathematics and Computer Science, Zhejiang A&F University, Hangzhou 311300, China

**Keywords:** CRISPR-Cas9, off-target prediction, deep learning, lightweight model, hyperparameter

## Abstract

Background: The CRISPR-Cas9 system has emerged as one of the most promising gene-editing technologies in biology. However, off-target effects remain a significant challenge. While recent advances in deep learning have led to the development of models for off-target prediction, these models often fail to fully leverage sequence pair information. Furthermore, as the models’ parameter sizes increase, so do their complexities, limiting their practical applicability. Methods: In this study, we introduce a novel multi-feature independent encoding method, which encodes the gRNA-DNA sequence pair into three distinct feature matrices to minimize information loss. Additionally, we propose a lightweight hybrid deep learning framework, CRISPR-MFH, that integrates multi-scale separable convolutions and hybrid attention mechanisms for efficient and accurate off-target prediction. Results: Extensive experiments across multiple benchmark datasets demonstrate that the proposed encoding method effectively captures critical features and that CRISPR-MFH outperforms or matches state-of-the-art models with significantly fewer parameters across multiple evaluation metrics. Conclusions: This study offers a novel perspective for advancing deep learning technology in the realm of CRISPR-Cas9 off-target detection.

## 1. Introduction

The CRISPR-Cas9 system, derived from the adaptive immune mechanisms of bacteria and archaea, emerges as a transformative tool in gene editing and is extensively applied in gene function studies, disease model development, gene therapy, and agricultural biotechnology [1,2,3,4]. This system is composed of CRISPR (Clustered Regularly Interspaced Short Palindromic Repeats) sequences and the Cas9 nuclease [5,6]. CRISPR sequences are characterized by short, repetitive DNA segments interspersed with spacer sequences derived from viral or plasmid DNA, enabling the CRISPR-Cas9 complex to specifically recognize and bind target DNA sequences. With guidance from a single guide RNA (sgRNA), the Cas9 enzyme introduces precise double-strand breaks (DSBs) at target DNA sites, facilitating gene knockout, repair, or insertion [7,8]. However, due to similarities between sgRNA-designed sequences and non-target regions in the genome, Cas9 exhibits some tolerance for mismatches between the sgRNA and DNA, particularly at the sgRNA’s 5^′^ end [9]. Consequently, while CRISPR-Cas9 can efficiently target specific DNA sequences, it may also cleave unintended, non-target DNA regions, causing off-target effects or unintended genetic modifications [10,11,12]. Addressing and accurately predicting these off-target effects are critical to expanding the safe and effective application of the CRISPR-Cas9 system.

Off-target detection methods for CRISPR-Cas9 are broadly categorized into biological experimental techniques and computational prediction approaches [13,14]. Biological methods utilize direct or indirect experimental processes to identify actual off-target sites within the genome [15]. Among site-specific methods, the T7 Endonuclease I assay (T7E1 assay) [16] and PCR amplification of predicted off-target sites are common, with verification conducted via Sanger sequencing [17] or deep sequencing [18]. However, these methods have limited sensitivity, making them less effective in detecting low-frequency off-target events. For genome-wide detection, methods such as GUIDE-seq (Genome-wide Unbiased Identification of DSBs Enabled by Sequencing) employ high-throughput sequencing to pinpoint integration sites, reducing interference from complex intracellular environments [19]. Digenome-seq (in vitro Cas9-digested whole-genome sequencing) operates independently of cellular factors, while CIRCLE-seq (Circularization for In vitro Reporting of Cleavage Effects by Sequencing) utilizes circularization to lower background noise, enhancing detection sensitivity and accuracy [20]. Additionally, SITE-seq (Selective Enrichment and Identification of Tagged Genomic DNA Ends by Sequencing) is highly sensitive, capable of detecting even low-frequency events [21]. Despite their effectiveness, these genome-wide methods are technically complex, time-consuming, and costly, requiring a high level of technical expertise. In light of these challenges, the development of computational prediction methods has gained momentum. Computational approaches offer a more accessible and efficient alternative, leveraging advancements in prediction algorithms to reduce reliance on extensive laboratory resources [22].

Computational methods for CRISPR-Cas9 off-target prediction can be categorized into three primary types: heuristic rules, machine learning models, and the more advanced deep learning approaches [23]. Early heuristic methods, based on empirical observations, consider factors like mismatch count, location, and distribution. Tools such as MIT [24] and CCTop [25], for instance, assess base mismatches between the sgRNA and potential off-target sites, the Protospacer Adjacent Motif (PAM) sequence, and set a maximum allowable number of mismatches. These tools operate on the core assumption that mismatches closer to the PAM sequence significantly affect Cas9 activity, producing an off-target score for each site. CROP-IT, an additional tool in this category, incorporates sequence characteristics and secondary structure for improved prediction accuracy [26]. Although these rule-based methods are valuable, they are limited by simplistic scoring systems and preset rules that cannot fully capture the complexity of off-target interactions, leading to restricted generalizability.

The development of machine learning has introduced more sophisticated prediction capabilities for CRISPR-Cas9 off-target effects [27]. Machine learning models, such as Elevation, utilize Gradient Boosting Decision Trees and leverage extensive CRISPR-Cas9 data while considering multiple sequence features, including base composition, GC content, secondary structure, and PAM types [28]. Another model, CRISPRedict, is a straightforward linear model for predicting sgRNA efficiency, offering accuracy comparable to advanced tools [29]. However, machine learning models often rely heavily on feature engineering and can struggle with the complexity of bioinformatics tasks. Additionally, they are less effective in handling high-dimensional, large-scale datasets, which limits their adaptability and scalability for more complex predictions [30].

Deep learning has become a driving force in bioinformatics, particularly in off-target prediction, due to its ability to automatically extract features and model complex nonlinear relationships, thereby achieving superior accuracy and adaptability. In off-target prediction for CRISPR-Cas9, these models first encode off-target sequences, followed by deep learning model applications. For instance, Lin et al. [31] introduced a CNN model for off-target prediction by encoding off-target sequences into a 23 × 4 matrix using one-hot encoding. Expanding on this, Liu et al. [32] incorporated word embeddings and positional encoding for sequences, which were then processed through a hybrid CNN and Transformer model for more refined predictions. To address indels in CRISPR-Cas9, Lin et al. [33] also devised an encoding method with an additional channel to represent indels, resulting in a 24 × 7 matrix to encode off-target pairs. Further advancements include Guan et al.’s [34] CRISPR-DNT model, which enhances prediction robustness by mitigating noise in training datasets. Luo et al. [35] leveraged the BERT model architecture to construct one of the largest off-target prediction models, integrating multiple encoding strategies to improve model interpretability. Sun et al. [36] developed a multi-view deep learning network specifically for sgRNA off-target effect prediction, setting a high standard in the field. Yang et al. [37] tackled class imbalance by introducing an ESB data rebalancing method, enhancing performance across diverse datasets. Despite these advancements, current methods still face limitations. Many overlook the cumulative impact of hyperparameters on model training when applied to datasets of varying sizes, which can significantly influence performance outcomes. Additionally, encoding strategies often compress sequence information, potentially leading to the loss of important details about the original sgRNA and target DNA. As model parameters increase, the expected gains in predictive performance have not always materialized, presenting practical challenges in deploying these models efficiently.

To enhance off-target prediction accuracy and address current limitations, this study introduces an encoding scheme that integrates sgRNA and target DNA into a unified encoding format while preserving distinct sequence encodings. This strategy generates a multi-feature input matrix that captures both unique sequence features and paired characteristics. The proposed model, CRISPR-MFH, is a lightweight, efficient architecture tailored to process multi-feature inputs. It employs a hybrid learning framework with multi-branch separable convolutions and multi-dimensional attention mechanisms, optimizing predictive performance while maintaining a streamlined structure. In addition, we examined the impact of two crucial hyperparameters—batch size and random seed—on off-target prediction outcomes. To improve interpretability, we implemented a controlled nucleotide transformation technique, enabling visual insights into feature recognition and emphasizing the significance of specific base positions and types. Experimental results across multiple datasets demonstrate that CRISPR-MFH achieves high accuracy and generalizability in off-target prediction tasks, validating its potential as a competitive approach.

The detailed plan and structure of this paper are as follows:

1. Section 2 provides an overview of the materials and methods used, including a description of the datasets and preprocessing procedures, the encoding strategy, and the architecture of the CRISPR-MFH model.
2. Section 3 presentS the experimental setup followed by a comprehensive analysis of the results. We begin by demonstrating the effectiveness of our model lightweighting strategy and then analyze the impact of various hyperparameters on model performance. A polynomial fitting method is proposed to determine two optimal hyperparameter values. Finally, we compare the performance of the proposed model with existing methods. Additionally, a base substitution approach is employed to investigate how different sequence features influence the model’s off-target prediction accuracy.
3. Finally, Section 4 concludes the paper, summarizing the key findings and discussing the limitations of the current model.

## 2. Materials and Methods

### 2.1. Datasets

In this study, we compile two types of publicly available datasets widely used in the field of off-target assays. The first type consists of seven datasets (D1–D7) containing only mismatches, while the second type includes two datasets (D8–D9) that contain both mismatches and insertions and deletions (indels). These datasets derive from different cell types using various site detection techniques. Each sample contains one sgRNA and its corresponding DNA sequence and label.

As presented in Table 1, a total of nine benchmark datasets were employed in this study. These datasets are categorized into two groups based on the types of off-target events they include: mismatch-only datasets (D1–D7), which consider only base mismatches between the sgRNA and DNA target sequences, and mismatch-and-indels datasets (D8–D9), which incorporate indels in addition to mismatches. Each dataset varies significantly in terms of the number of sgRNAs used, THE detection methods applied, and the balance between positive and negative samples.

D1 is collected using SITE-Seq, an in vitro detection method that operates independently of a reference genome. This dataset comprises NINE sgRNAs and over 217,000 sequence pairs, among which 3767 are labeled as active off-target sites. D2 to D4 integraTE results from various hybrid detection techniques, including BLESS, PCR, Digenome-Seq, and HTGTS. These datasets involve 49 sgRNAs in total and contributE approximately 300,000 samples, with 708 of them being confirmed as positive off-target events. D5–D7 utilize GUIDE-Seq, a widely adopted high-throughput and highly sensitive technique for in vivo off-target site detection. These datasets involve 36 unique sgRNAs and produce a large number of potential off-target candidates (>970,000), but only a small fraction (464) are confirmed as active sites. These datasets present significant class imbalance, posing a challenge for model training and evaluation.

D8 is generated using CIRCLE-Seq, which is capable of detecting off-target sites containing insertions and deletions. This dataset includES 10 sgRNAs and over 577,000 sequence pairs, with 7371 being identified as positive cases, making it one of the most informative resources for indels-aware prediction tasks. D9 also relIES on GUIDE-Seq and contains SIX sgRNAs, along with 213,883 negative and 50 positive samples. While the number of positive sites is limited, this dataset offeRS critical validation for models designed to handle both mismatch and indels patterns.

Overall, these datasets provide a diverse and challenging benchmark for modeling off-target activities. The variability in detection technologies, sgRNA designs, and imbalance ratios allows for rigorous evaluation of model robustness and generalization across different biological and experimental conditions.

### 2.2. Data Analysis

An effective way to avoid off-target effects in CRISPR-Cas9 is to maximize cutting efficiency while minimizing off-target occurrences, which requires achieving both high specificity and sensitivity. To elucidate the factors influencing off-target effects, we analyze the sequence characteristics within the original off-target dataset.

**Figure 1:**
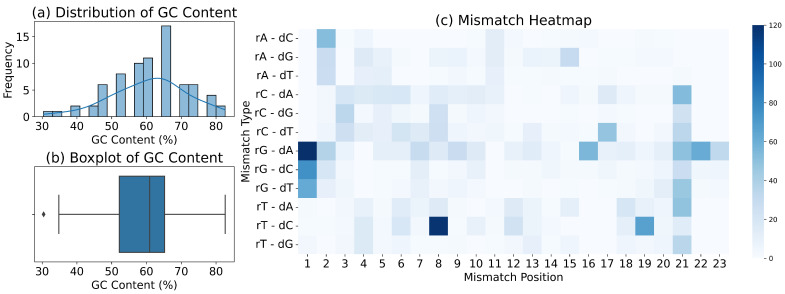
Analysis of GC content, base mismatch location and type, and data imbalance in the dataset.

We first examine the GC content across all active off-target sites. GC content plays a dual role by influencing both the thermal stability of the DNA sequence and the binding efficiency of the Cas9-sgRNA complex. Regions with high GC content are more prone to forming secondary structures, which impair the binding affinity of sgRNAs to Cas9 and subsequently increase the likelihood of off-target events. As illustrated in Figure 1a,c, the GC content of the 23 bp sequences in most active off-target sites is predominantly above 50%, with some reaching as high as 90%. The peak GC content is approximately 65%, and the median value, as indicated by the box plot, exceeds 60%, aligning with previous studies. In addition, to ensure that our coding method and model capture the nuances of mismatch types and positions, we analyze the distribution of mismatches across all off-target sites. As depicted in Figure 1b, the position of the mismatch significantly influences off-target activity, with mismatches occurring at the distal end from the PAM (positions 1–8 dp) being more prevalent. This suggests a higher tolerance for mismatches at the distal end compared to the proximal end. In terms of mismatch types, the most prominent impact on off-target activity occurs with guanine base mismatches, particularly G-A mismatches at the 1dp position. Finally, we analyze the distribution of positive and negative samples across all datasets and identify a significant issue of data imbalance. Three of the datasets exhibit Imbalance Ratios (IRS) exceeding 1000, with the maximum value reaching 6846, categorizing them as heavily imbalanced datasets. Imbalance ratios and GC content are calculated as follows:

(1) $$GC\;\mathrm{Content}=\frac{G+C}{N}$$

where *N* denotes the total number of bases in the sgRNA (i.e., the length of the sequence). *G* indicates the number of bases of guanine in sgRNA; *C* indicates the number of bases of cytosine.

(2) $$IR=\frac{N_{major}}{N_{minor}}$$

where $N_{major}$ denotes the number of samples in the majority category in the dataset and $N_{minor}$ denotes the number of samples in the minority category in the dataset.

### 2.3. Encoding sgRNA-Target DNA Sequences

The off-target effects of CRISPR-Cas9 are influenced not only by the binding affinity between the sgRNA and the target DNA but also by the inherent characteristics of both the sgRNA and target sequences. These characteristics encompass factors such as sequence composition, nucleotide ratios, and RNA secondary structures. Notably, the GC content of sgRNAs significantly affects their cleavage activity; sequences with higher GC content generally exhibit greater stability and cleavage efficiency, which results in better target binding and cleavage efficiency. Thus, incorporating these features into the encoding process is crucial for the effectiveness of model training.

**Figure 2:**
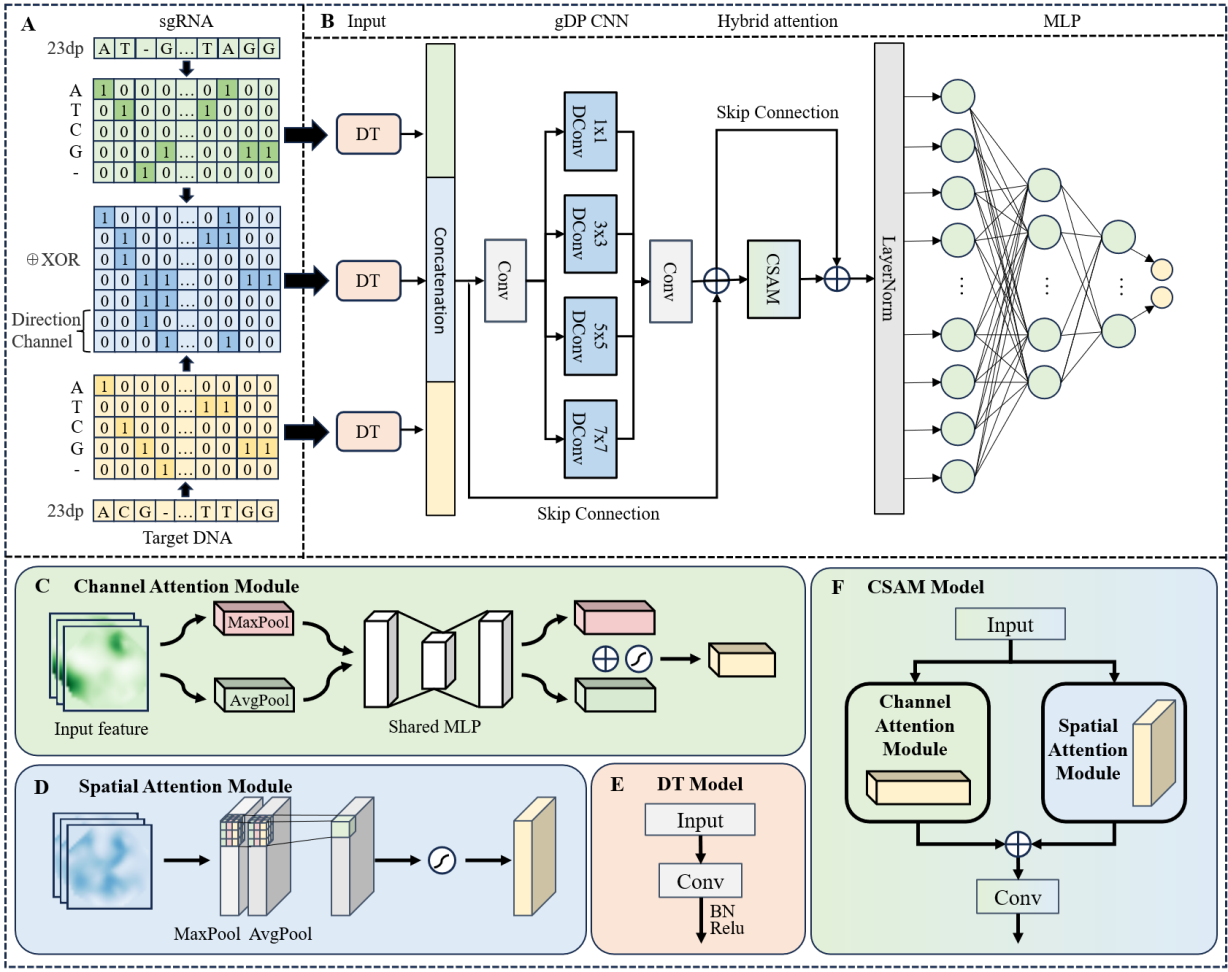
(**A**) Data encoding process, the green part is the gRNA encoding, the orange part is the DNA encoding, and the blue part is the fusion encoding after the OR operation. (**B**) Model structure diagram of CRISPR-MFH. (**C**) Structural diagram of the channel attention model in the model. (**D**) Structural diagram of the spatial attention model in the model. (**E**) Structural diagram of DT. (**F**) Structural diagram of CSAM.

When encoding sequences, it is essential to consider multiple feature perspectives. As illustrated in Figure 2A, we initially employed the one-hot encoding method to separately encode sgRNA and target DNA, resulting in feature matrices of dimensions 23 × 5. In this encoding scheme, base A is represented as [1,0,0,0,0,0], base T as [0,1,0,0,0,0], and so on for other bases. This method effectively transforms discrete base information into a numerical format suitable for deep learning models, while the resulting feature matrices encapsulate information regarding sequence composition. To address datasets that include indels, we introduced a new channel (_) to represent these features, which is particularly significant in gene editing contexts, as indels can alter gene function.

Subsequently, we conducted an allosteric operation on these two feature matrices to create a new matrix that effectively captures the unique features arising from the binding interactions between sgRNAs and target sites. This approach allows for a structured and detailed representation of genetic information. It is important to note that the mismatch positions become indistinguishable following the heterozygous operation; for instance, the mismatch pairs ‘TC’ and ‘CT’ are both encoded as [0,1,1,0,0]. To resolve this issue, we added two additional channels, [0,1] and [1,0], to differentiate the directional context, thereby preserving original features and preventing information loss. This design not only enhances model accuracy but also effectively captures the interactions between sgRNA and target DNA. Ultimately, these three feature matrices will be concurrently input into the model for training, thereby improving predictions of off-target effects.

### 2.4. Model Construction

In previous studies, Long Short-Term Memory (LSTM) [42] and Transformer [43,44] models have commonly been employed to predict off-target effects by processing DNA base sequences. However, these models were initially designed for handling long sequences, such as those found in natural language processing tasks. Given that RNA sequences typically consist of only 23 base pairs, their short length limits the ability of LSTM and Transformer models to fully exploit long-range dependency mechanisms, which constrains their effectiveness in this context [45]. Moreover, the complexity of these models results in inefficient training, especially when working with smaller datasets typical in biological research. Another challenge is the high parameter count in LSTM and Transformer models, which, when combined with limited training data, impairs their ability to effectively learn essential biological features [46].

In this study, we decompose the sequence pairs into three distinct feature matrices. Simultaneously, to efficiently capture the diverse features within the sequences while maintaining a lightweight architecture, we propose a novel hybrid learning framework called Multi-branch Feature Hybrid Learning Networks (CRISPR-MFH), specifically designed for off-target prediction tasks. The CRISPR-MFH architecture, shown in Figure 2B, consists of four key components: the Dimensional Transform (DT) module, the Grouped Depthwise and Pointwise Convolution (gDP) module, the Channel-Spatial Attention Mechanism (CSAM), and a Multi-Layer Perceptron (MLP) module, each targeting a specific aspect of feature extraction and prediction.

The model’s input comprises three feature matrices encoded from the gRNA-Target DNA sequences. These matrices are passed into the Dimensional Transform (DT) module, shown in Figure 2E, which includes a convolutional layer, Batch Normalization, and a ReLU activation function. The DT module first applies a convolutional layer to perform linear transformations over the input sequence in a sliding-window manner, enabling the extraction of local pattern information between adjacent bases and the learning of low-level features representing diverse base combinations. Following this, Batch Normalization is employed to mitigate issues such as gradient explosion and vanishing gradients, while also enhancing the model’s robustness to variations in hyperparameters. Subsequently, a ReLU activation function is introduced to inject nonlinearity into the network, thereby endowing it with the capacity to approximate complex nonlinear functions.

Importantly, the DT module not only achieves dimensional alignment across the input feature matrices but also incorporates parameterized feature extraction at the earliest stage of the network. This ensures that subsequent modules—such as the gDP and CSAM—receive inputs that are both dimensionally consistent and rich in structural information. Compared to the naive concatenation of raw inputs, this approach yields superior representational efficiency and semantic expressiveness, thereby contributing to improved overall model performance.

The three processed features are concatenated to form a fusion feature matrix, which is then processed through the gDP module. Depthwise separable convolution significantly reduces computational complexity by separately extracting correlations between feature channels and spatial dimensions, making it far more efficient than standard convolutional operations. In our gDP, we employ a four-branch depthwise separable convolution, using convolutional kernels of sizes 1 × 1, 3 × 3, 5 × 5, and 7 × 7. The larger kernels capture fine-grained, detailed features over a broad spatial range, while the smaller kernels focus on lower-resolution semantic information. To further enhance the model’s robustness, we utilize skip connections that add the processed deep convolution output back to the input. This preserves the original features, mitigates the vanishing gradient problem, and improves the overall training efficiency of the network. This leads to a more comprehensive feature extraction process, achieving a balance between computational efficiency and predictive accuracy. The depthwise separable convolution output is calculated as follows:

(3) $$Out=X+\mathrm{concat}\left(\sum_{k_{i}\in 1,3,5,7}\mathrm{Conv}dw\left(X,k_{i}\right)\right)$$

where *X* is the original input feature. Convdw is a depth-separable convolution operation with four different sizes. $k_{i}$ represents different deep convolution kernel sizes. + is a residual concatenation, which adds the inputs to the result that has been convolved.

Subsequently, the features resulting from the gDP are input into the hybrid attention mechanism known as the CSAM model. Attention mechanisms have emerged as a critical component in deep learning, particularly in addressing sequence-based tasks [47]. Given that the proximity of bases to the PAM significantly impacts off-target effects, attention mechanisms allow models to effectively focus on essential information within the sequence matrix, capturing the relationships among the bases and identifying key regions in the sequence [48,49]. The CSAM comprises two parallel branches: channel attention and spatial attention. As shown in Figure 2C, the channel attention primarily extracts global features through Global Average Pooling (GAP) and Global Max Pooling (GMP), after which these features are weighted and fused via a shared fully connected layer. The attention weights for each channel are subsequently generated using the Sigmoid activation function. The mathematical formulation for the generation of these attention weights is presented below:

(4) $$CA=\sigma \left(\mathrm{MLP}(\mathrm{GAP}(X))+\mathrm{MLP}(\mathrm{GMP}(X))\right)$$

where CA is the channel attention. *X* is the input feature. GAP is the global average pooling. GMP is the global maximum pooling, and σ is the Sigmoid activation function.

As shown in Figure 2D, the spatial attention mechanism generates a spatial attention map by applying average pooling and maximum pooling operations over the channel dimensions of the input features. These pooled features are then concatenated to form a combined representation, which is subsequently processed through a 7 × 7 convolutional layer to generate the final spatial attention map. The corresponding formula for this operation is presented below:

(5) $$SA=\sigma \left(f^{7\times 7}\left([\mathrm{AvgPool}(X);\mathrm{MaxPool}(X)]\right)\right)$$

where SA is the spatial attention and $f^{7\times 7}$ denotes a 7 × 7 convolution.

As shown in Figure 2F, the CSAM module integrates both channel and spatial attention mechanisms. Compared to traditional fully convolutional networks, the CSAM module introduces the attention mechanism with fewer parameters, ensuring the module remains lightweight. By weighting the input features across both channel and spatial dimensions, the module significantly enhances the model’s capacity to capture expressive and relevant features. Additionally, jump connections are employed during implementation, where the original input features are summed with the attention-weighted features. This strategy effectively improves the network’s generalization ability. The mathematical formulation for this process is provided below:

(6) $$CSAM={\mathrm{Conv}}_{1\times 1}\left([CA\cdot X;SA\cdot X]\right)$$

CA·X: denotes channel attention reweighted input features, SA·X: denotes spatial attention reweighted input features, and convolution is applied to integrate the results after channel and spatial attention.

The MLP module functions as the final classification component of the CRISPR-MFH architecture. Following the convolutional and attention-based feature extraction stages—including the DT, gDP, and CSAM modules—the resulting high-level representations are input into the MLP for nonlinear transformation and decision-making. The MLP consists of three fully connected layers with progressively reduced dimensionality (e.g., 80, 20, and 2 neurons), where the final layer utilizes a Softmax activation function to output the probability scores for binary classification (i.e., off-target vs. non-off-target). To mitigate overfitting—particularly in light of class imbalance—a dropout layer with a rate of 0.35 is applied between layers during training. Although lightweight compared to preceding modules, the MLP plays a pivotal role in aggregating and refining the extracted features, mapping them into a compact and discriminative representation space. Ultimately, it serves as the decision-making head that determines the model’s final predictive output.

### 2.5. Performance Evaluation

Due to the significant imbalance in our dataset, it is imperative to select appropriate evaluation metrics that accurately reflect the performance of our proposed model and facilitate a reliable comparison with other models [50]. The prevalence of negative samples in the dataset—manifested by a large IR—leads to a model biased toward learning features of negative samples, consequently diminishing its predictive capability for positive samples. To address this, we have chosen to utilize a suite of evaluation metrics, including PR-AUC (Precision–Recall Area Under Curve), ROC-AUC (Receiver Operating Characteristic Area Under Curve), and Recall.

ROC-AUC, while commonly deployed, may not adequately reflect the model’s performance on positive samples due to its sensitivity toward the abundant negative samples. In the ROC curve, the True Positive Rate (TPR) forms the vertical axis and the False Positive Rate (FPR) the horizontal. Given the disproportionate number of negative samples, a model primarily predicting negatives could still achieve a low FPR, resulting in misleadingly optimistic ROC-AUC scores. In contrast, the PR curve, which plots Precision against Recall, offers a more focused evaluation of positive sample detection. This is particularly useful when positive samples are rare, as the PR curve more accurately demonstrates the model’s effectiveness in distinguishing between positive and negative classes. Specifically, PR-AUC provides a more direct measure of the model’s discriminative power under conditions of class imbalance. Precision and Recall are calculated as follows:

(7) $$Precision=\frac{\mathrm{TP}}{\mathrm{TP}+\mathrm{FP}}$$

(8) $$Recall=\frac{\mathrm{TP}}{\mathrm{TP}+\mathrm{FN}}$$

where Precision denotes the proportion of samples that are actually positive out of all samples predicted as positive by the model. TP denotes True Positive and FP denotes False Positive. Recall indicates the proportion of all samples that are actually positive that are correctly predicted to be positive. FN indicates False Negative.

Finally, the output of the hybrid attention module is passed into an MLP consisting of three layers with 80, 20, and 2 neurons in each layer. The last layer employs a softmax activation function, and a dropout rate of 0.35 is applied to randomly discard neurons, thereby mitigating overfitting.

### 2.6. Experimental Settings

To ensure the reproducibility of all experiments, we initialize the randomness of libraries such as TensorFlow and NumPy by fixing random seeds. For fair model training across all comparison experiments, we apply the same training parameters, environment, and hardware. All comparison models are implemented using the open-source code provided in the original papers. To standardize the environment, we employ the TensorFlow 2.3.2 deep learning framework along with two RTX 2080 Ti 22GB graphics cards. Notably, we found that GPUs above the 30 series do not support CUDA 11.2 due to driver limitations, which can cause model training to fail to converge; therefore, the TensorFlow version is kept below 2.4.

Given the imbalance between positive and negative samples, we apply a five-fold cross-validation approach for model training, ensuring that all samples serve as both training and test sets at different stages, thereby effectively evaluating the stability and reliability of the model results. Since models with varying numbers of parameters may require different convergence times during training, we set the epoch limit to 500 epochs and employ the EarlyStopping technique to terminate training early if the loss value remains unchanged for 10 consecutive epochs.

## 3. Result

### 3.1. Comparison of Model Complexity

As the application of deep learning in genomic data analysis continues to advance, the complexity and parameter count of models have shown a significant upward trend. We analyzed and compared the parameters and sizes of models released in recent years, including our proposed CRISPR-MFH model.

**Figure 3:**
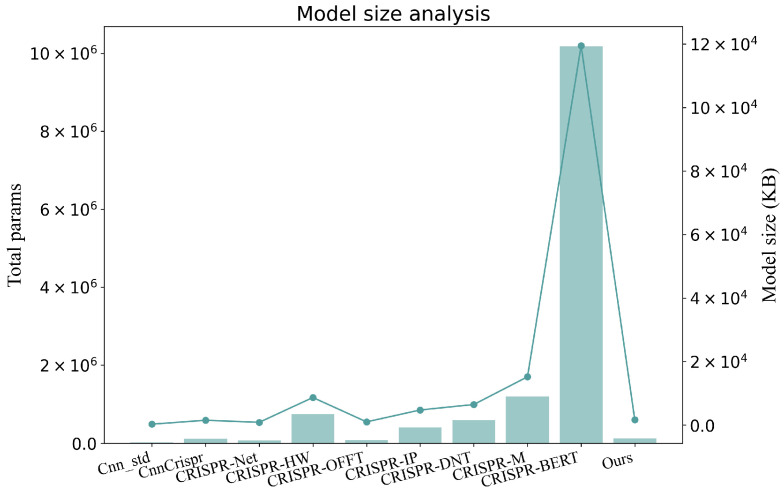
Parameters and model size of off-target prediction models in recent years.

As depicted in Figure 3, the earliest deep learning model, Cnn_std, used for CRISPR-Cas9 off-target prediction, consists of only 19,111 parameters, with a trained model size of merely 300 KB, making it the most lightweight model to date. In contrast, the CRISPR-M model released in 2024 has 1,196,417 parameters and a size of 15,219 KB, while the most recent CRISPR-BERT model surpasses 10 million parameters, reaching a size of 119,501 KB. However, the large scale of these models also poses challenges in terms of computational resources and practical applications, especially in the training and inference phases.

In comparison, our CRISPR-MFH model offers a more practical solution under resource constraints, with only 125,030 parameters and a trained model size of 1677 KB. This positions it between CnnCrispr and CRISPR-OFFT in terms of parameter count and model size while outperforming these models. The results indicate that CRISPR-MFH strikes a balance between off-target prediction performance and computational efficiency, delivering better accuracy than smaller models without consuming the vast resources required by larger models like CRISPR-BERT. Overall, CRISPR-MFH offers an optimal trade-off among model size, computational speed, and accuracy.

### 3.2. Effect of Hyperparameters on Off-Target Prediction

In this section, we explore the impact of deep learning model training hyperparameters on CRISPR-Cas9 off-target prediction, focusing on two key parameters: batch size (Bz) and random seed. Both parameters directly affect how the data are partitioned and, consequently, influence model performance and evaluation given the dataset’s imbalance. To ensure the generalizability of our experiments, we modified the CRISPR-MFH model by replacing all of its existing structures with an LSTM architecture, retaining only the coding component.

#### 3.2.1. Batch Sizes

**Figure 4:**
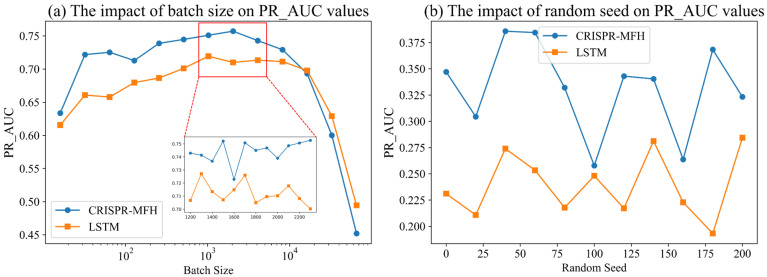
(**a**) PR_AUC values come from CRISPR-MFH and LSTM models that train with different batch sizes on the D4 dataset. (**b**) Using different random seeds affects the model’s off-target prediction.

As shown in Figure 4a, we record the average PR-AUC values for the five-fold cross-validation of the two models at different batch sizes on the D4 dataset. The batch size (Bz) is increased incrementally from 16 to 65,536 to identify the interval where the highest PR-AUC value is achieved. The experimental results show that the PR-AUC initially increases with Bz but then decreases as Bz reaches extreme values (e.g., 32,768 and 65,536). For the CRISPR-MFH model, the optimal PR-AUC value of 0.7571 occurs at a batch size of 2048, while the LSTM model reaches a similar optimal PR-AUC value in this range.

After identifying the optimal batch size, additional experiments are conducted with batch sizes between 1200 and 2300. Although PR-AUC fluctuations are minimal in this range, a local optimum can still be identified. The results indicate that when batch sizes are smaller (e.g., 16 to 512), the PR-AUC gradually increases. This improvement happens because larger gradient fluctuations allow the model to discover better parameter update paths. However, the optimal PR-AUC is not reached with smaller batch sizes due to the nature of the D4 dataset, which contains a large sample size but relatively few positive samples. With smaller Bz values, it becomes challenging to include enough positive samples in each batch, making it difficult for the mean and variance of the positive samples to represent the overall data distribution, thus increasing error rates.

Furthermore, when the batch size exceeds 8192, the PR-AUC starts to decline significantly. This drop likely occurs because the stability associated with large batch sizes causes the model to rely too much on local gradients, reducing the learning rate and potentially leading to overfitting, particularly in datasets with limited positive samples. Based on these observations, we propose a polynomial model to calculate the optimal batch size for off-target prediction, which is determined by the following formula:

(9) $$y_{2}=8.35\times 10^{-8}\cdot x^{2}+0.0042\cdot x-278.33$$

(10) $$y_{3}=-2.43\times 10^{-13}\cdot x^{3}+1.99\times 10^{-7}\cdot x^{2}-0.01\cdot x+79.84$$

(11) $$Bz_{\mathrm{final}}=round\left(\frac{y_{2}+y_{3}}{2}\right)$$

where *x* is the sample size of the dataset. $y_{2}$, $y_{3}$ are the predicted batch sizes of the quadratic and cubic polynomials.

#### 3.2.2. Random Seeds

The random seed influences weight initialization, dataset segmentation, and the gradient descent process in deep learning, leading to fluctuations in model performance. We conduct an experiment to investigate the effect of random seeds on PR-AUC values using the D6 dataset. By adjusting the random seeds, we observe and record the PR-AUC values of two models. As shown in Figure 4b, the PR-AUC value of CRISPR-MFH exhibits significant variation under different random seeds, with the lowest value observed at a seed of 100 (PR-AUC: 0.2578) and the highest at a seed of 40 (PR-AUC: 0.3858). These results demonstrate that the model is sensitive to variations in training data caused by different weight initializations and dataset divisions.

Different random seeds may result in divergent gradient descent paths, impacting the solution to which the model ultimately converges. Additionally, since we apply PR-AUC as an evaluation metric, which is particularly sensitive to the model’s overall performance, changes in random seeds that affect data division (such as the split between training and validation sets) are likely the primary cause of this fluctuation. In subsequent experiments, we find that the effect of random seeds on model predictions decreases as the sample size of the dataset increases. Therefore, to enhance the stability of the model and ensure fairness in model comparisons, we address this issue by averaging results over several experiments, fixing the seed, and standardizing the weight initialization method.

### 3.3. Ablation Experiment

In this section, we assess the individual contributions of each component within our CRISPR-MFH model toward off-target prediction by conducting a series of ablation experiments using dataset D5. As detailed in Table 2, the first step involves modifying the input layer by removing the two independent feature matrices from the encoding module, resulting in ablation model 1 (AM1). Next, the one-hot encoding of the two independent sequences is replaced with word-vector encoding to form ablation model 2 (AM2). Subsequently, we systematically freeze key modules in the model, including the CSAM, gDP, and MLP modules, yielding ablation models AM3, AM4, and AM5, respectively. This approach allows us to isolate and examine the impact of these individual components on the overall performance of the model.

From the experimental results, it is evident that the off-target prediction performance of the original CRISPR-MFH model on dataset D5 achieves a PR-AUC of 0.5823, a ROC-AUC of 0.9941, and a Recall of 0.5212. In AM1, where the two independent feature matrices in the encoding part are removed, the PR-AUC decreases by 2.4% to 0.5678, and Recall drops significantly by 12.1%, reaching 0.4578. This substantial reduction in Recall highlights the critical role of retaining the separate feature matrices in capturing distinct sequence features, which significantly contributes to the model’s accuracy. Similarly, for AM2, where One-hot encoding is replaced with word-vector encoding, further reductions are observed, with PR-AUC falling to 0.5505 and Recall to 0.4505. These results suggest that one-hot encoding, along with the independent encoding of gRNA and DNA sequences, plays a pivotal role in accurately capturing unique sequence characteristics essential for improving model precision.

In AM3, where the CSAM is frozen, the PR-AUC decreases by 4.2% to 0.5574, and Recall sees a significant 8.1% drop to 0.4788. This underscores the importance of the CSAM module in identifying key bases within sequences and enabling the model to capture long-range dependencies—factors critical for off-target prediction. In AM4, where the gDP module is omitted, further performance degradation occurs, with PR-AUC decreasing to 0.5327 and Recall dropping to 0.4399. This suggests that the gDP module is vital for extracting spatial features across different scales, optimizing computational efficiency, and balancing performance with complexity. Lastly, in AM5, where the MLP is removed, there is a modest decline in performance, with PR-AUC dropping to 0.5460 and Recall to 0.4754. However, the impact is less severe than the removal of the CSAM or gDP modules, indicating that while the MLP contributes to refining feature representations, its absence has a relatively smaller effect than the more critical roles played by the attention mechanism and convolutional components.

### 3.4. Comparison with State-of-the-Art Models

In this section, we evaluate the superiority of our CRISPR-MFH model in off-target prediction by comparing it to nine existing off-target prediction models using five-fold cross-validation on mismatch datasets. For this comparison, we select two datasets, a large dataset (D1) and a small dataset (D2), both containing only mismatches. Additionally, to demonstrate the model’s versatility and effectiveness on datasets with indels events, we conduct further experiments on the D8 dataset.

For model selection, we compare our CRISPR-MFH model with nine existing mismatch off-target prediction models. Among these, CRISPR-MCA and CRISPR-BERT are the most recently proposed models, with CRISPR-BERT currently having the largest number of parameters. CNN_std, as the first model to apply deep learning techniques to off-target prediction, serves as a representative baseline. CRISPR-DNT introduces a novel approach by incorporating noise handling into a deep learning model for the first time. CRISPR-OFFT and CnnCrisp are among the earliest models to employ word vector encoding for off-target prediction. For models that also include predictions on indels datasets, CRISPR-Net is notable for being the first to consider the effects of sequence bulges, while CRISPR-IP specifically encodes bulges to account for these variations. This selection of comparison models allows for a comprehensive evaluation of CRISPR-MFH’s performance across different approaches to off-target prediction.

#### 3.4.1. Comparison of Different Models on Mismatch-Only Datasets

**Figure 5:**
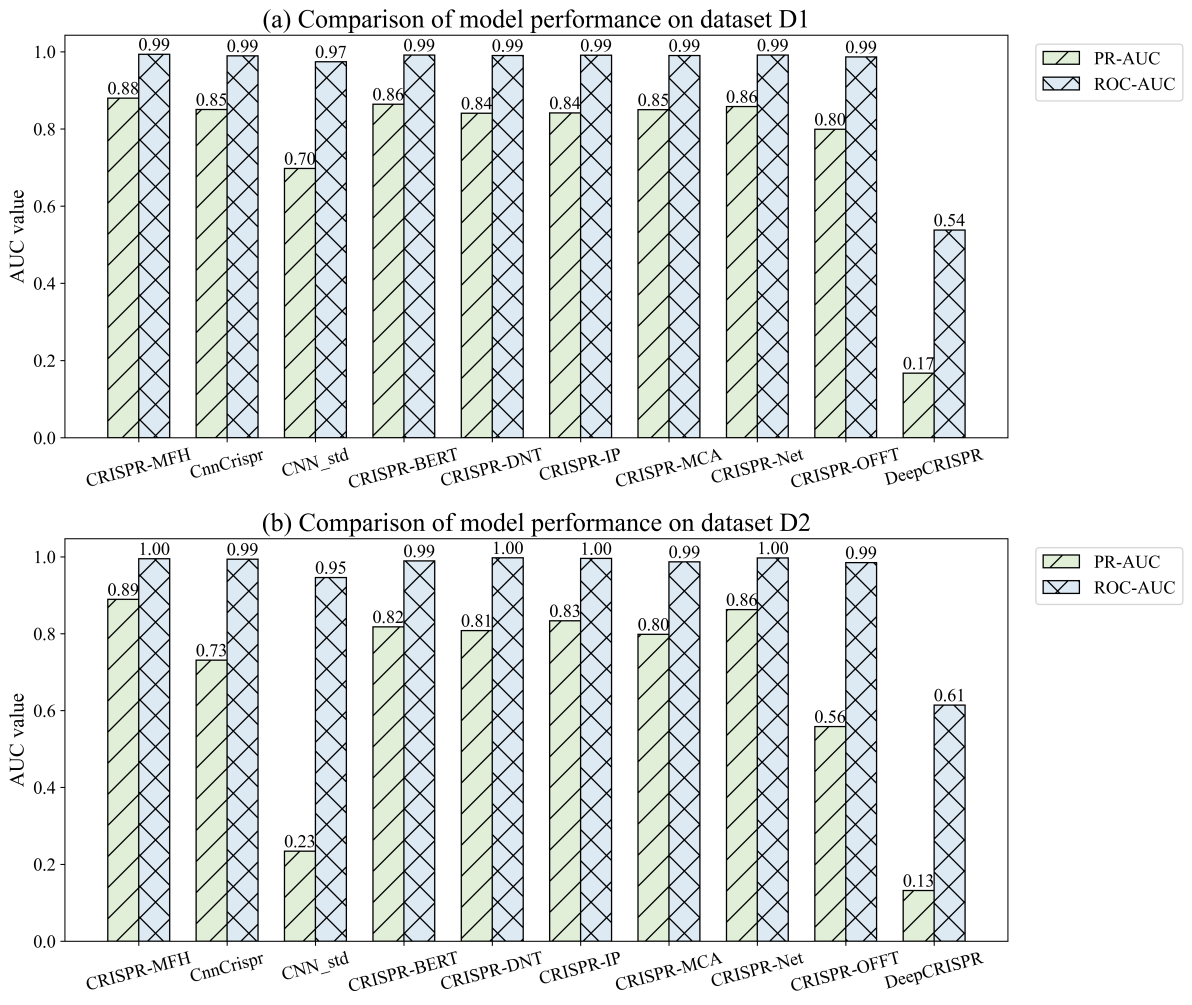
Comparison of off-target predictions of existing models on mismatch-only datasets D1 and D2.

We first perform a five-fold cross-validation on the large dataset D1, as shown in Figure 5. The average PR_AUC of the CRISPR-MFH model is 0.88, which is 2.3% and 25.7% higher than the similarly lightweight models CRISPR-Net (0.86) and CNN_std (0.70), respectively. It also shows a 3.5% and 2.3% improvement over the recently proposed CRISPR-MCA (0.85) and the more complex CRISPR-BERT model (0.86), respectively. The DeepCRISPR model, due to its oversimplification, struggles to learn the features of the off-target dataset, resulting in a significantly lower performance of 0.17. On the ROC_AUC metric, all models except DeepCRISPR achieve higher values of 0.97 or more, likely due to the imbalance of the dataset. Additionally, on the small dataset D2, the model’s predictive ability is particularly tested. Our model achieves the optimal PR_AUC value of 0.89, which is 3.4% better than the next-best CRISPR-Net (0.86) and represents an 8.5% improvement over the recently released CRISPR-BERT model. Since the dataset has a small number of positive samples, the values of ROC_AUC are all above 0.99 except for DeepCRISPR, and no further comparative analysis is needed. Overall, the superior predictive performance of our model on both large and small datasets demonstrates its high stability and accuracy.

#### 3.4.2. Comparison of Different Models on Datasets Containing Both Mismatches and Indels

**Figure 6:**
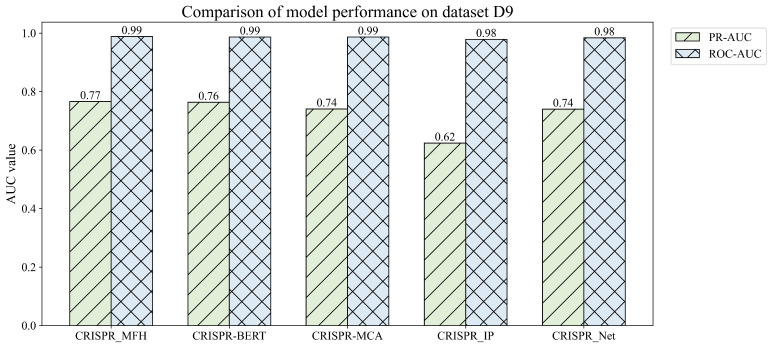
Comparison of off-target predictions of existing models on containing both mismatches and indels datasets D9.

Given that sgRNAs containing indels are capable of exhibiting cleavage activity, we assessed our model’s performance using the D8 dataset. As illustrated in Figure 6, the proposed lightweight model, CRISPR-MFH, achieved a PR_AUC score of 0.77, surpassing the performance of the CRISPR-BERT model, despite the latter’s significantly higher parameter count. Notably, CRISPR-MFH demonstrated a 4.1% improvement over both CRISPR-MCA and CRISPR-Net, and a remarkable 24.2% increase in PR_AUC compared to CRISPR-IP, which attained a score of 0.62. These findings indicate that CRISPR-MFH is highly effective in predicting off-target activities, including those associated with both mismatches and indels events.

### 3.5. Comparison of Generalizability

**Figure 7:**
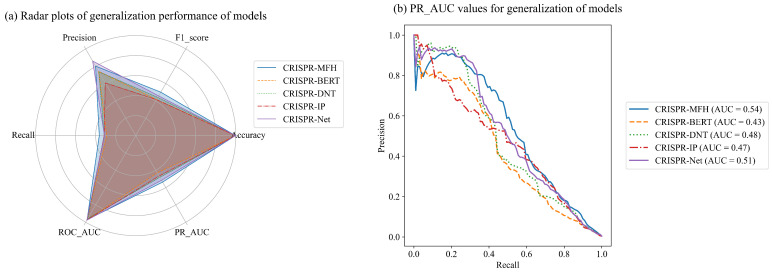
Comparison of off-target predictions of existing models on dataset D9 containing both indels and mismatch. (**a**) Radargram containing six metrics. (**b**) PR_AUC for five models on the D9 dataset.

To assess the model’s robustness on unseen data and its adaptability across various off-target prediction tasks, we conduct generalization experiments comparing the CRISPR-MFH model with other leading models. All models are trained on the D5–7 dataset, generated through genome-wide detection using GUIDE-Seq, and are subsequently validated on the smaller D2 dataset across multiple evaluation metrics. Figure 7a,b present a radar chart illustrating the comparative performance of each model. Our model achieves the highest scores across several metrics, particularly in PR_AUC (0.54) and F1 score (0.50).

All models demonstrate exceptionally high accuracy rates, surpassing 0.996, with the CRISPR-MFH and CRISPR-NET models leading at 0.997. While accuracy is a useful metric, relying on it exclusively can be misleading in CRISPR datasets due to class imbalance, which may mask important performance aspects. The F1 score, which balances precision and recall, provides a more informative metric in this context. CRISPR-MFH shows the strongest F1 score (0.50), outperforming CRISPR-NET (0.46) by 8.7%. Other models yield F1 scores around 0.4, underscoring the benefit of more streamlined models. In terms of PR_AUC, the larger CRISPR-BERT model achieves only 0.43, indicating that while heavily parameterized models may excel on a specific dataset, they might struggle to capture critical features in off-target datasets, limiting their efficacy in predicting position-based data.

### 3.6. Visualization of CRISPR-MFH on the gRNA-DNA Target Prediction

To assess the ability of CRISPR-MFH in capturing the key features of off-target datasets, we employed a base control variable approach. In this method, each base in the 23 bp target sequence is substituted individually, and the resulting changes in off-target scores are quantified. The off-target score difference is defined as the difference between the original off-target score of the base pair and the score after base substitution. This difference serves as an indicator of the model’s sensitivity to base changes, reflecting how specific base substitutions influence the predicted off-target effects. To ensure robustness and stability, we generated 10,000 PAM sequences, all terminating with the common PAM sequence “AGG”, under identical conditions used in the CRISPR-M model. For each sequence, every base position was substituted with one of the other three possible nucleotides or an indel. As a result, we analyzed a total of 10,000 × 23 × 4 samples, providing a comprehensive evaluation of the model’s performance across various base substitutions.

**Figure 8:**
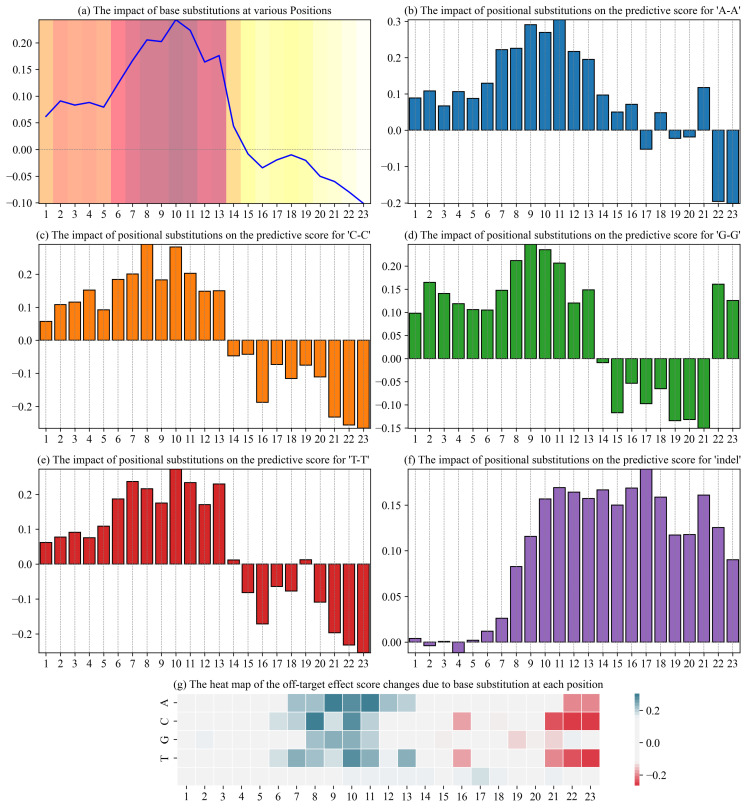
Visualization plot of CRISPR-MFH off-target predicted sensitivity. The x-axis of each plot base pair positions, and the y-axis is the mean value of score changes induced by the nucleotide control variable method. (**a**) Change in score due to base substitutions at each position. (**b**–**f**) Results of sensitivity analysis for single base substitutions at A-A, T-T, G-G, C-C and indel. (**f**) These sensitivity scores from the five bars are aggregated into a heat map. (**g**) Heat map of sensitivity scores due to base substitutions.

Figure 8a illustrates the overall impact of base substitutions on the predicted off-target score at each position. It is evident from the figure that substitutions in positions 1 to 10 result in a significant positive shift in the predicted score, indicating that these regions are particularly sensitive to base changes. However, from position 11 onwards, the impact of base substitutions gradually diminishes, with a negative shift becoming more pronounced by position 15. Notably, base substitutions closer to the PAM region (positions 16 to 23) tend to have a negative effect on the predicted score. This observation aligns with the CRISPR-Cas9 mechanism, where the PAM sequence, located downstream of the target sequence (typically at the 3^′^ end), plays a critical role in Cas9 binding. Substitutions near the PAM sequence are more likely to disrupt Cas9’s ability to bind efficiently to the target DNA, which has been observed in other off-target prediction models [51,52,53]. CRISPR-MFH typically assigns higher weights to features in the PAM-proximal regions, where structural alterations are more likely to influence Cas9 binding and cleavage efficiency.

Figure 8b–f present the effects of specific base substitutions on the predicted off-target scores. Figure 8b shows the impact of A-A substitutions at each position. A notable observation is that A-A substitutions in positions 3 to 13 lead to a significant positive impact on the predicted score, with the greatest effect observed between positions 8 and 12. This could be attributed to the simpler molecular structure of adenine, which facilitates the formation of hydrogen bonds and enhances Cas9 binding. Furthermore, in the first half of the sequence, A substitutions are less likely to disrupt the DNA secondary structure, a factor that deep learning models often capture, resulting in an increase in the predicted off-target score. Figure 8c–e illustrate the effects of C-C substitutions. In the first half of the sequence (positions 3 to 7), C-C substitutions demonstrate a strong positive impact on the predicted score, similar to the A-A substitutions. However, starting from position 12, C-C substitutions begin to exert a negative effect on the predicted score, suggesting that cytosine (C) substitutions in the earlier positions promote binding stability, while those in the later positions may disrupt binding. This pattern is consistent with the model’s sensitivity analysis. Figure 8f depicts the impact of indels. Indels, especially those occurring after position 10, lead to a marked increase in the predicted score, with the peak effect around position 15. Indels can significantly alter the structural integrity of the target sequence, which is detected by the model as structural anomalies, thus increasing the off-target score. Moreover, indels often introduce mismatches in the Cas9 binding site, which further contributes to the predicted off-target effects. This highlights the importance of considering indels in off-target prediction, as they can substantially influence binding affinity and cleavage efficiency [54,55,56].

Figure 8g shows the correspondence between the peak region observed in Figure 8a (positions 6–13) and the heatmap from Figure 8g, further validating that the model captures key sequence features related to base substitutions. This alignment indicates that the CRISPR-MFH model effectively identifies the impact of base substitutions on off-target effects, particularly in the regions surrounding the PAM sequence. These findings underscore the utility of the base control variable analysis method in revealing how base pair substitutions in the gRNA target sequence influence the predicted off-target activity.

Overall, the results demonstrate that the base control variable approach provides valuable insights into the structural and functional roles of different bases at various positions within the target sequence. This approach not only enhances our understanding of the underlying mechanisms of off-target effects but also contributes to the optimization of gRNA design.

## 4. Conclusions

Off-target effects in CRISPR-Cas9 represent a major obstacle to its widespread application. Developing accurate and computationally efficient off-target prediction methods is essential to addressing this challenge. This study was driven by two central hypotheses: (1) that gRNA–DNA sequence pairs contain rich, position-dependent information essential for precise off-target prediction and (2) that a carefully designed lightweight model can achieve high predictive performance while minimizing computational overhead.

In response to these hypotheses, we proposed CRISPR-MFH, a novel hybrid deep learning framework that integrates multi-scale depthwise separable convolutions and a dual attention mechanism, specifically tailored for off-target prediction. A key innovation lies in our multi-feature independent encoding scheme, which captures positional, categorical, and physicochemical characteristics of gRNA–DNA interactions. This encoding design significantly mitigates information loss and enhances the model’s ability to extract biologically relevant sequence patterns.

Comprehensive experiments on both mismatch-only and mismatch-plus-indel datasets demonstrate that CRISPR-MFH outperforms existing models across multiple evaluation metrics, while maintaining a significantly lower parameter count. Ablation studies further confirm the effectiveness of each architectural component, particularly the encoding module. Additionally, we employed a base control variable method to interpret the model’s learned representations, revealing biologically meaningful sensitivities at specific base positions—especially near PAM sites—thus providing mechanistic insight into off-target cleavage behavior.

To address challenges posed by data imbalance and variable dataset sizes, we further proposed a novel use of polynomial regression to estimate optimal batch sizes and conducted a sensitivity analysis on random seeds to understand their influence on training stability and model performance.

Despite the strong empirical results, we acknowledge that current training data are largely derived from in vitro human cell line assays, which may introduce system-specific biases. To enhance the generalizability of CRISPR-MFH, we recommend fine-tuning the model with domain-specific datasets when applied to different species, tissues, or clinical scenarios. In future work, we aim to (1) incorporate epigenetic and chromatin accessibility features to model regulatory context; (2) adapt the framework to accommodate other gene editing tools, such as base editors and prime editors; and (3) integrate uncertainty quantification techniques to improve the reliability of predictions in high-stakes biomedical applications.

In summary, CRISPR-MFH offers a biologically grounded, computationally efficient, and extensible solution for CRISPR off-target prediction. By combining a modular encoding strategy with a lightweight yet expressive architecture, it advances the development of safer and more reliable genome editing technologies.

## Figures and Tables

**Table 1:** Details of the two types of datasets utilized in the experiments and analyses.

Type	Dataset	Detection Method	sgRNA	PS	NS	IR
Mismatch-only	D1 [21]	SITE-Seq	9	3767	213,966	56
	D2 [38]	Hybrid detection	12	120	20,199	168
	D3 [39]	Hybrid detection	19	52	10,077	193
	D4 [38]	Hybrid detection	18	536	132,378	246
	D5 [19]	GUIDE-Seq	9	354	294,180	831
	D6 [40]	GUIDE-Seq	5	54	95,775	1773
	D7 [28]	GUIDE-Seq	22	56	383,407	6846
Mismatch and indels	D8 [41]	CIRCLE-seq	10	7371	577,578	78
	D9 [28]	GUIDE-Seq	6	50	213,883	4277

IR: Imbalance ratio; PS: Positive sample; NS: Negative sample.

**Table 2:** Comparison of ablation experiments and coding schemes.

Model	PR-AUC	ROC-AUC	Recall
CRISPR-MFH	0.5823	0.9941	0.5212
AM1	0.5678	0.9930	0.4578
AM2	0.5505	0.9869	0.4505
AM3	0.5574	0.9934	0.4788
AM4	0.5327	0.9927	0.4399
AM5	0.5460	0.9943	0.4754

AM1: Without individual coding; AM2: Replacing two sequences encoded as the word vector approach; AM3: Without CSAM; AM4: Without gDP; AM5: Without MLP.

## Data Availability

Supporting datasets and source codes for this study are readily accessible at https://github.com/Zhengyanyi-web/CRISPR-MFH (accessed on 26 March 2025).
